# Health literacy and oral health-related behaviors among young adults in Norway

**DOI:** 10.2340/aos.v84.44230

**Published:** 2025-08-14

**Authors:** Lene Marita Steinvik, Gro Eirin Holde, Hanne S. Finbråten, Elin K. Evensen, Linda Maria Stein

**Affiliations:** a Department of Clinical Dentistry, Faculty of Health Sciences, UiT The Arctic University of Norway, Tromsø, Norway; b The Public Dental Health Service Competence Centre of Northern Norway, Tromsø, Norway; c Department of Health and Nursing Sciences, Faculty of Social and Health Sciences, University of Inland Norway, Elverum, Norway; d Department of Health and Care Sciences, Faculty of Health Sciences, UiT The Arctic University of Norway, Tromsø, Norway; e Oral Health Centre of Expertise in Rogaland, Stavanger, Norway

**Keywords:** Health literacy, preventive dentistry, public health dentistry, oral hygiene, dental health services

## Abstract

**Objective:**

The transition from adolescence to adulthood represents an increase in autonomy and responsibility of oral health-related behaviors, in which health literacy might play a significant role. The objective of the study was to assess health literacy in a young adult population and determine whether health literacy is associated with their oral health-related behaviors.

**Materials and methods:**

Utilizing data from the Fit Futures longitudinal cohort study, this cross-sectional analysis was based on self-administered questionnaires from the third wave, FF3. Health literacy was assessed using a short version of the European Health Literacy Survey Questionnaire (HLS-Q12), while oral health-related behaviors were assessed through toothbrushing frequency and dental service utilization. In addition, socioeconomic factors were included as control variables. Bivariate analyses and multivariable logistic regression were performed.

**Results:**

The findings indicated that 38% of the young adults had a score equivalent to having lower levels of health literacy. Higher health literacy scores were associated with more regular dental service use, even after adjusting for socioeconomic covariates.

**Conclusion:**

Although most young adults had good oral health-related behaviors, it is concerning that a significant proportion still neglects regular dental visits and consistent tooth brushing. These findings highlight the need for greater emphasis on health literacy within public dental health services.

## Introduction

The transition from adolescence to adulthood includes several key milestones and transitional events, such as finishing school, leaving the family home, starting college or university studies, starting a career and entering working life, forming relationships, and becoming a parent [1]. In terms of oral health, this transition entails increasing autonomy in oral health-related behaviors, and simultaneously, shifting the responsibility for maintaining oral health-promoting behavior [2]. Oral health-related behavior comprises practices that prevent oral diseases and maintain good oral health, including oral hygiene practices, diet, avoiding harmful habits, and regular use of dental services [3]. During this transitional period into adulthood, some young adults might experience difficulties in managing their oral health, understanding preventive measures like tooth brushing, and seeking appropriate dental care [4–6].

In Norway, the Public Dental Health Service emphasizes preventive oral health care through national guidelines and promotes regular use of dental services, reflecting the strong preventive focus shared among Scandinavian countries [7, 8]. The Public Dental Health Service primarily offers free-of-charge dental screening and treatment for children until the age of 18 [9]. This is an outreaching offer constituted by national law; but after the age of 18, the responsibility shifts. Young adults are then required to independently find, contact and utilize dental services on their own, as well as pay for them out of pocket, like the general adult population. However, if these preventive efforts, such as encouraging tooth brushing twice daily according to national guidelines, or regular use of dental services, are carried through by the young adults into adulthood remains unknown. A study on delay of dental care among young adults suggested that health literacy may be an overlooked factor influencing oral health-related behaviours in this age group [10].

Health literacy is defined as ‘people’s knowledge, motivation and competencies to access, understand, appraise, and apply health information in order to make judgments and take decisions in everyday life concerning healthcare, disease prevention and health promotion to maintain or improve quality of life during the life course’ [11]. Previous research has established that higher levels of health literacy are linked to positive health outcomes, including improved self-care practices and use of health services [12, 13]. Conversely, individuals with lower levels of health literacy often experience poorer health outcomes due to difficulties in understanding health instructions, adhering to preventive measures, and effectively communicating with healthcare providers [12, 14].

Adults with lower health literacy have been found to have less favorable oral health-related behaviors, like eating more sugary foods [15], less frequent tooth brushing [16], missing more dental appointments, and using dental services primarily for acute issues [17, 18]. While research on the association between health literacy and oral health-related behaviors exists, further research is needed, particularly in young adulthood. Nonetheless, studies indicate that improving health literacy is achievable and can lead to better oral health behaviors and outcomes, highlighting its potential as a target for oral health interventions [19–21].

The Public Dental Health Service in Norway has been designed to mitigate social disparities by giving children equal opportunity to achieve oral health. However, oral health-related behaviors still follow a social gradient, being closely linked to socioeconomic status and the social determinants of health [22]. According to the World Health Organization (WHO), health literacy is recognized as a social determinant of health, comparable to socioeconomic factors such as education, income, and employment [23]. Hence, socioeconomic factors must be accounted for when studying the association between health literacy and oral health-related behaviors [14, 22].

The shift into taking personal responsibility for oral health-related behaviors and use of dental services requires health literacy. A recent Norwegian national survey found that 33% of the adult population could be considered having low health literacy [24]. The high prevalence of low health literacy is not only a concern in Norway but also a widespread international issue [23]. However, evidence suggests that young adults are disproportionately affected [24, 25]. Young adults need to be able to access, understand, appraise, and apply health information to prevent oral diseases and to maintain good oral health throughout their lives. Furthermore, they might struggle to achieve this if their health literacy levels as a collective are low.

Accordingly, this study aimed to assess health literacy in a young adult population and determine whether health literacy is associated with their oral health-related behaviors.

## Materials and methods

### Study design

**Figure 1:**
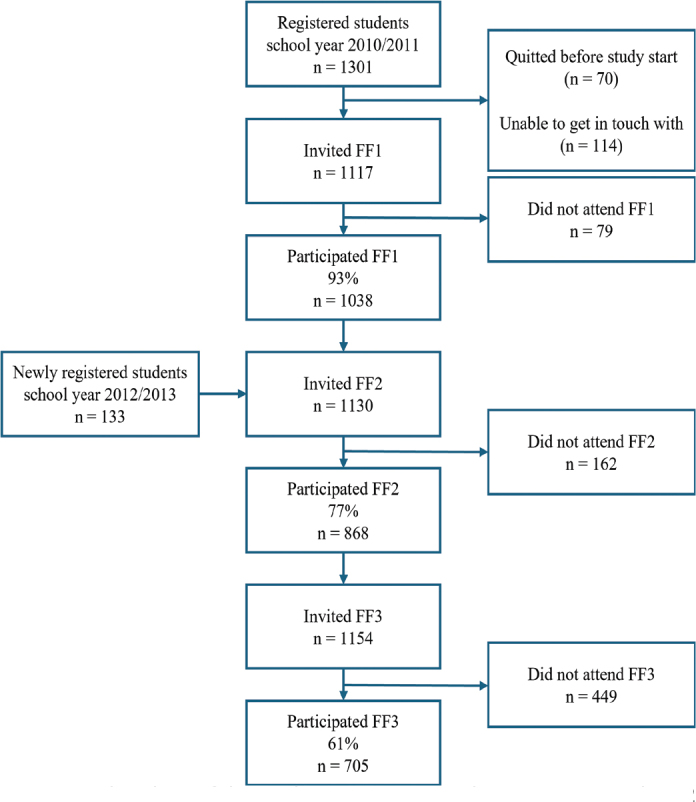
Flowchart of the study participation in the Fit Futures study 1, 2 and 3.

The present study is a part of the Fit Futures study, a population-based health study that aims to increase knowledge about young people’s health and lifestyle. The Fit Futures study has a longitudinal design, including a cohort of adolescents from Tromsø and neighboring, more rural, municipalities in Northern Norway over a 10-year period [26]. Fit Futures 1 (FF1) was conducted in 2010–11, and all students in their first year of upper-secondary school during this school year were invited (*n* = 1117), in which 1038 participated (92.9%, mean age 16.4, standard deviation [SD] ±1.2, 49.0% female). The first follow-up study, Fit Futures 2 (FF2), was performed 2 years later, with no clinical oral health data. A third wave, Fit Futures 3 (FF3), was conducted in 2021–22, 10–11 years after the first wave, where 705 participated in FF3 (61.1%, mean age 26.9, SD ±1.2). Please see Figure 1 for details. The collected data included multiple self-administered questionnaires and extensive clinical health examinations, including oral examinations and collection of biological samples. The present study used data from the third wave (FF3) acquired from the self-administered questionnaires.

All participants provided written informed consent before participating in the Fit Futures studies. The regional committee for medical and health research ethics approved the Fit Futures study (reference number 2009/1282) and the present study (reference number 563627). In addition, the present study was reviewed by SIKT – the Norwegian Agency for Shared Services in Education and Research – with no objections to the data management (reference number 660941).

### Measures

Health literacy was assessed using a short version [27] of the European Health Literacy Survey Questionnaire [28] (HLS-Q12), which reflects Sørensen’s definition and conceptual model of health literacy [6]. The HLS-Q12 consists of 12 items dealing with how difficult it is to access, understand, appraise, and apply health information within health care, disease prevention and health promotion settings (Table S1). The items offer four response alternatives, ‘very difficult (1)’, ‘difficult (2)’, ‘easy (3)’ and ‘very easy (4)’, where a higher score indicates higher health literacy. In addition, the participants had the option of selecting ‘don’t know’, which was categorized as missing. Participants with more than three missing items were excluded from the analysis. The scale has been validated in a Norwegian population and is considered a feasible instrument for measuring health literacy [27]. The HLS-Q12 sum score ranges from 12 to 48, and was dichotomized at the defined level 1 [24], where participants with a score ≤ 33 would indicate level 1 or ‘lower health literacy’ and scores > 33 indicate level 2/3 or ‘higher health literacy’.

Oral health-related behaviors included self-reported tooth brushing frequency and use of dental services. Tooth brushing frequency was assessed by asking, ‘How often do you usually brush your teeth?’. The alternatives were ‘Less than once a week’, ‘A few times a week’, ‘Once daily’ or ‘2 or more times a day’. Tooth brushing frequency was further dichotomized according to the national Norwegian oral health guidelines for children and adults advocating tooth brushing twice daily with fluoride toothpaste [7], where participants who brushed ‘Less than once a week’, ‘A few times a week’ and ‘Once daily’ were grouped together as ‘< Twice daily’, and ‘2 or more times a day’ remained unaltered as ‘≥ Twice daily’.

Use of dental services was assessed by asking, ‘Do you have regular visits to dental services?’, where the participants could answer ‘No, never’, ‘No, just when I have acute problems’, ‘Yes, more than two years apart’, ‘Yes, every second year’, ‘Yes, every year’ or ‘Yes, more than once a year’. This variable was dichotomized according to whether the participant answered ‘Yes’ regardless of frequency and ‘No’. Further, participants who answered ‘No, never’, ‘No, only acute’, or ‘Yes, more than two years apart’ received an additional question regarding their reasoning behind irregular use of services. The participants could choose between the statements, ‘Have not felt the need for a dental visit’, ‘Have not prioritized dental visits’, ‘Afraid to go and see a dentist or dental hygienist’, ‘Economic reasons’, or ‘Other reasons’. The question about the use of dental services was not included at baseline in FF1 as all participants were eligible for regular dental screenings provided by the Norwegian Public Dental Health Service [9].

Predisposing characteristics, such as age and gender, were recorded. Socioeconomic factors included educational level, work participation, and financial situation.

Educational level was measured by asking participants, ‘What is the highest education you have completed?’, where participants could answer ‘Primary education’, ‘Occupational high school’, ‘High school’, ‘University less than 4 years’, or ‘University 4 years or more’. This variable was categorized into three categories, where primary education remained unaltered, secondary education included both ‘Occupational high school’ and ‘High school’, and higher education included both ‘University less than 4 years’ and ‘University 4 years or more’.

Work participation was assessed by asking participants, ‘Do you work/study?’, where participants could answer ‘Full-time’, ‘Part-time’, ‘Student’, ‘Parental leave’, ‘Sick leave’, ‘Unemployed’, ‘Work assessment allowance’, ‘Disabled’ or ‘Social welfare’. Participants could choose more than one alternative. Based on work participation, responses were grouped into three categories. Working full-time (1) included those who responded ‘full-time’. Partially working (2) included those who responded ‘Part-time’, ‘Student’, ‘Parental leave’ and ‘Sick leave’. Not working (3) included those who responded ‘Unemployed’, ‘’Work assessment allowance’, ‘Disabled’ or ‘Social welfare’.

Financial situation was assessed by asking participants ‘How do you evaluate your finances today?’. The question could be answered on a Likert scale (1–5), where the participants could indicate their financial situation as ‘Very Poor’, ‘Fair’, ‘Average’, ‘Good’ or ‘Excellent’. The variable was categorized for the analysis into three categories, where the answers ‘Very Poor’ and ‘Fair’, and the answers ‘Good’ and ‘Excellent’ were merged into two categories, ‘Poor’ and ‘Good’ respectively. The alternative ‘Average’ remained unaltered.

### Statistical analyses

Statistical analyses were performed using IBM SPSS Statistics version 29. Data from all participants who participated in FF3 were included in the analyses. Statistical significance was set at *p* < 0.05.

Missing data occurred at a very low frequency for all the included questionnaire data (0–3.8 %). Further analysis of these participants suggested that the missing data pattern appeared randomly. All missing cases were excluded pairwise in the analyses. For the HLS-Q12 scale, the missing frequency was 16.6%. This included 91 participants with more than three missing items (12.9%). These participants were rather heterogenous in terms of gender, socioeconomic factors and oral health-related behaviors, and excluding these participants from the analyses did not change the variance of HLS-Q12, nor the predictability in the multivariable analysis, by more than one decimal place. However, as the total score of HLS-Q12 is a sum score of all 12 items combined, including these participants would not correctly identify their level of health literacy. Therefore, they were excluded from the analyses. Furthermore, at the item level for HLS-Q12, the missing frequency varied from 3.9 to 22.6%, where item 8 (*decide how you can protect yourself from illness using advice from family or friends*) had the highest missing frequency. The internal consistency of the HLS-Q12 was acceptable (Cronbach’s alpha of 0.77). To assess the distribution of health literacy across the oral health-related and socioeconomic variables, bivariate analyses were performed for the dichotomized health literacy variable. Bivariate analyses consisted of cross-tabulations and Pearsons Chi-Square tests (χ^2^).

A logistic regression model was built with use of dental services as the outcome. Health literacy along with gender, educational level, work participation and financial situation was entered as covariates. The assumptions for multivariable logistic regression were controlled and satisfied (Tolerance value > 0.9 and Variance Inflation Factor (VIF) < 1.4, indicating no multicollinearity). Furthermore, the correlation matrix displayed no correlations exceeding *r* = 0.7 among the included variables, further indicating no issues with multicollinearity.

## Results

### Sample characteristics

Sample characteristics from FF3 are presented in Table 1. In FF3, there was a higher proportion of females (55%) than the first wave, FF1. The age ranged from 26 to 36 years, where 93.5% was in the age range of 26–28. The majority of participants in FF3 reported that they had completed a higher educational level than secondary or primary education. Further, most participants worked full-time and considered their financial situation average or good as young adults. Yet a small proportion of the young adults had not completed secondary education, did not participate in working life and considered their financial situation poor.

**Table 1:** Sample characteristics from FF3.

Sample characteristics	FF3 (age 26–36)	
	*n* (%)	Missing *n* (%)
**Total**	705 (100)	
**Age, mean (SD)**	26.9 (1.2)	0 (0)
**Gender**		0 (0)
Female	388 (55.0)	
Male	317 (45.0)	
**Educational level**		26 (3.7)
Primary education	41 (5.8)	
Secondary education	266 (37.7)	
Higher education	372 (52.8)	
**Work participation**		27 (3.8)
Working fulltime	399 (56.6)	
Partially working	233 (33.1)	
Not working	46 (6.5)	
**Financial situation**		26 (3.7)
Poor	89 (12.6)	
Average	251 (35.6)	
Good	339 (48.1)	
**Tooth brushing frequency**		26 (3.7)
Twice daily or more	488 (69.2)	
Once daily	156 (22.1)	
Few times a week or less	35 (5.0)	
**Use of dental services**		26 (3.7)
Yes	507 (71.9)	
No, never or only acute	172 (24.4)	


FF3: Fit Futures 3; SD: standard deviation.

### Health literacy distribution

The distribution of health literacy among participants as young adults is presented in Table 2. Of the 705 participants who returned for FF3, 588 (83.4%) completed the HLS-Q12 questionnaire. The scores ranged from 20 to 48 with a median score of 35. The mean score of the HLS-Q12 was 35.3 (SD ±6.0), and visual inspection indicated a normal distribution of scores with a 5% trimmed mean equal to the mean score. The findings indicated that 37.9% had a health literacy level below or equal to 33, equivalent to the defined level 1 [24, 29]. Participants with a lower health literacy level tended to report lower educational levels and poorer perceived financial situations. Participants with higher health literacy levels tended to report higher education, working full-time and perceived their financial situation as good more frequently.

**Table 2:** Health literacy distribution and bivariate associations between health literacy, socioeconomic covariates and oral health-related behavior from FF3.

Health literacy distribution	HLS-Q12 *n*	Lower HL ≤ 33 *n* (%)	Higher HL > 33 *n* (%)	*p* *
**Total**	588	223 (37.9)	365 (62.1)	
**Gender**				0.159
Female	338	120 (35.5)	218 (64.5)	
Male	250	103 (41.2)	147 (58.8)	
**Educational level**				0.001
Primary education	30	19 (63.3)	11 (36.7)	
Secondary education	220	93 (42.3)	127 (57.7)	
Higher education	338	111 (32.8)	227 (67.2)	
**Work participation**				0.134
Not working	36	19 (52.8)	17 (47.2)	
Partially working	204	79 (38.7)	125 (61.3)	
Working fulltime	348	125 (35.9)	223 (64.1)	
**Financial situation**				< 0.001
Poor	76	43 (56.6)	33 (43.4)	
Average	219	91 (41.5)	128 (58.5)	
Good	293	89 (30.4)	204 (69.6)	
**Tooth brushing frequency**				0.096
< Twice daily	159	69 (43.4)	90 (56.6)	
≥ Twice daily	429	154 (35.9)	275 (64.1)	
**Use of dental services**				<0.001
No, never or only acute	143	74 (51.7)	69 (48.3)	
Yes	445	149 (33.5)	296 (66.5)	

* Differences between groups assessed with Pearson χ^2^.

FF3: Fit Futures 3; HL: Health Literacy; HLS-Q12: Short version of the European Health Literacy Survey Questionnaire.

### Tooth brushing frequency

About two-thirds reported brushing their teeth twice daily or more frequently in FF3 (Table 1). When comparing health literacy levels for the young adults in FF3, participants with higher health literacy levels tended to report tooth brushing twice daily or more, more frequently than those with a lower health literacy level (Table 2). The bivariate analyses did not indicate a significant difference in health literacy levels according to tooth brushing frequency among the young adults in FF3.

### Use of dental services

A majority of participants reported regular use of dental services as young adults, while approximately one-fourth reported irregular use of dental services or only dental visits in cases of acute issues (Table 1). When asked about the reasoning behind irregular use of dental services, the most frequent response was economic reasons (31.4%), followed by prioritization (28.4%), perceived need (25.1%), fear (12.4%) and other reasons (2.7%). When comparing health literacy levels, a higher percentage of participants with higher health literacy had regular use of dental services than participants with lower health literacy (Table 2). Bivariate analyses indicated significant associations between use of dental services and gender, educational level, work participation, and financial situation. Therefore, these variables were included in the multivariable logistic regression (Table 3).

**Table 3:** The association between health literacy and regular use of dental services, presented as Pearson χ^2^, and odds ratios (OR) and 95% confidence intervals (CI) derived from the multivariable logistic regression models.

	Pearson χ^2^	Unadjusted analysis			Adjusted analysis		
	*P*	OR	95% CI	*p*	OR	95% CI	*p*
**Health literacy (HL)**	< 0.001						
(ref. Lower HL ≤ 33)		1			1		
Higher HL > 33		2.1	1.4–3.1	< 0.001	1.8	1.2–2.6	0.006
**Gender**	0.029						
(ref. Female)		1			1		
Male		0.7	0.5–0.9	0.030	0.8	0.5–1.1	0.163
**Educational level**	< 0.001						
(ref. Primary education)		1			1		
Secondary education		1.8	0.9–3.5	0.089	1.3	0.6–2.9	0.579
Higher education		3.2	1.6–6.3	< 0.001	1.7	0.8–4.0	0.202
**Work participation**	< 0.001						
(ref. not working)		1			1		
Partially working		2.7	1.4–5.2	0.003	1.6	0.7–3.6	0.256
Working fulltime		2.7	1.4–5.0	0.002	1.1	0.5–2.6	0.751
**Financial situation**	0.005						
(ref. Poor)		1			1		
Average		1.8	1.1–3.1	0.018	1.9	1.0–3.5	0.036
Good		3.0	1.8–5.0	< 0.001	2.8	1.4–5.4	0.002

Note: Nagelkerke *R*^2^ for the adjusted analysis = 0.09.

CI, confidence interval; HL, health literacy; OR, odds ratio.

The multivariable logistic regression for having regular use of dental services is presented in Table 3. The analyses revealed an association between use of dental services and health literacy, even when controlling for the effect of socioeconomic covariates. Participants with higher health literacy had 2.1 higher odds of using dental services regularly than those with lower health literacy, and this association was slightly reduced when adjusting for socioeconomic variables. For the socioeconomic covariates, financial situation was positively associated with use of dental services, indicating that participants who considered their financial situation as good had higher odds of regular use of dental services than partici-pants who considered their financial situation as poor.

## Discussion

The current findings indicated that many young adults may struggle with accessing, understanding, appraising, and applying health information, seemingly affecting their oral health-related behaviors. In this sample, 38% of young adults had lower health literacy, slightly above the national adult average of 33% but consistent with the 37% reported among 16- to 24-year-olds [24]. These findings align with research identifying young adulthood as a critical period for health literacy [25]. Their lower health literacy as a group may relate to their limited experience with illness or their perceived need for preventive measures. Keeping in mind that health literacy is considered a dynamic ability and is adaptable throughout the life course [11, 14], young adults have the possibility to improve both health literacy and oral health-related behavior preventively. Therefore, health literacy can be seen as both a potential risk factor and a resorce in the maintenance of oral health [20].

Furthermore, higher health literacy levels were associated with having regular use of dental services, implying that lower health literacy was associated with irregular and more acute use of dental services. These findings are supported by existing literature [17, 18]. For tooth brushing frequency, the findings are consistent with previous research presenting various inconclusive findings [16, 30]. The current findings suggest that oral health-related behaviors are influenced by a range of social determinants, rather than health literacy alone. Accordingly, the explained variance (*R*^2^) in the multiple models was low. As indicated by previous research on young individuals, oral health and oral health-related behaviors are multifaced, influenced by both individual, lifestyle, social, and environmental factors [31]. Change in beliefs and socioeconomic factors seem to be a part of the transition into adulthood in relation to oral health-related behavior [4]. Health literacy might be a part of this maturation and affect young adults’ oral health over time.

The oral health-related behaviors seem to remain good during the transition into young adulthood. The young adult participants would have received regular, free-of-charge dental screenings and treatment, including preventive measures like chair-side education for 18+ years. Their persistent good oral health-related behaviors as a group may have had an impact on their overall oral health and well-being. Compared to the first wave, FF1, there was a reduction in the percentage of participants who reported tooth brushing a few times a week or less from 13% to 5%. This indicates that a small proportion in this sample continued to exhibit poor oral health-related behaviors as young adults. This might suggest that the measures traditionally used in public dental health services are not targeted enough. Interventions and effective communication about dental health practices and preventive care can be tailored to various demographic groups and health literacy levels to improve overall health literacy and better oral health [14, 21].

The Norwegian Ministry of Health and Care Services published in 2019 a national strategy to enhance health literacy across the population [32]. This strategy focuses on healthcare personnel, decision-makers, and leaders within healthcare and social care services, as well as patient and user organizations. It advocates for a combination of individual- and system-level approaches, emphasizing the necessity of tailoring messages and communication to the target audience’s specific needs. Implementing the national health literacy strategy in the Norwegian Public Dental Health Service might be an essential step to achieving more equal services. Hence, providing accessible and understandable oral health information when young people are using dental services, and including more use of upstream measures outside the dental clinic is important.

The Fit Futures study is a unique dataset with high participation rates, giving much needed insight into health and oral health during the transition into adulthood. However, a prominent limitation of this study is only including the HLS-Q12 and use of dental services in the follow-up study, FF3. This limits the comparability of both health literacy levels and use of dental services over time. However, during the planning and collecting data for the first wave, FF1, the importance of health literacy was yet to be discussed. Furthermore, as adolescents in FF1, the participants would have received an outreaching, regular offer of dental services – free of charge [9].

The use of the HLS-Q12 in this study was determined by its inclusion in the Fit Futures survey, which was a general health survey. Although domain-specific instruments may offer greater content validity for assessing oral health literacy (e.g. REALD-30 or HeLD [33, 34]), these were not available in the dataset, nor appropriate for the Fit Futures study scope. The HLS-Q12 captures core competencies relevant to oral health-related behaviors, such as accessing, understanding, and applying health information – highly relevant to oral health behaviors and decision making. Given that health and oral health are deeply interconnected, the use of HLS-Q12 in this study can be considered appropriate. Nonetheless, the limited validation of the HLS-Q12 in younger populations and oral health contexts represents a limitation of the study.

Misclassification from categorization and cut-offs may be a potential source of bias. These procedures were necessary for the analyses but might result in losing some variance in the sample. For instance, the categorization of health literacy scores from the HLS-Q12 was originally developed based on data from computer-assisted telephone interviews [29], and therefore the cut-off value might not be directly transferable when using online self-administered questionnaires. Furthermore, the participants had the option to select ‘don’t know’, whereas during the original interviews this option was reserved for the interviewer only [27]. While making the option of selecting ‘don’t know’ more available, this may have resulted in a higher missing frequency for HLS-Q12. These remarks need to be considered when reviewing the current findings.

The Fit Futures cohort-sample included students attending their first-year upper secondary school, excluding those that were not enrolled or attending. This might have led to some underrepresentation of individuals only completing primary education. Additionally, the slight overrepresentation of female participants in the follow-up indicated some attrition among male participants. It is also important to note that individuals with the lowest health literacy may also struggle to complete self-administered questionnaires, potentially leading to an overrepresentation of health-literate young adults. Furthermore, when interpreting self-reported oral health-related behaviors, it is also important to consider the potential influence of social desirability bias, where participants may respond according to social norms rather than their actual behaviors.

## Conclusion

A considerable number of young adults had lower health literacy, which was related to less optimal oral health-related behaviors. Higher health literacy scores were associated with more regular dental service use, and this association remained significant after adjusting for socioeconomic covariates. Although most young adults had good oral health-related behaviors, it is concerning that a significant proportion still neglects regular dental visits and consistent tooth brushing. Future research should investigate whether the association with health literacy can be transferable to the oral health of young adults. Regardless, it is eminent that health literacy needs more consideration in public dental health services.
